# Multifunctionality and Processability of a Thermoplastic Based Gel Electrolyte Cell for the Realization of Structural Batteries

**DOI:** 10.1021/acs.jpcc.4c07301

**Published:** 2024-12-10

**Authors:** Martin Krammer, Susan Montes, Helmut Kühnelt, Qixiang Jiang, Daniel Lager, Alexander Bismarck, Alexander Beutl

**Affiliations:** 1 Center for Transport Technologies, Battery Technologies, AIT Austrian Institute of Technology GmbH, Giefinggasse 2, Vienna 1210, Austria; 2 Center for Transport Technologies, Electric Vehicle Technologies, AIT Austrian Institute of Technology GmbH, Giefinggasse 2, Vienna 1210, Austria; 3 Polymer and Composite Engineering (PaCE) Group, Insitute of Materials Chemistry & Research, Faculty of Chemistry, University of Vienna, Währinger Str. 42, Vienna 1090, Austria; 4 Center for Energy, Sustainable Thermal Energy Systems, AIT Austrian Institute of Technology GmbH, Giefinggasse 2, Vienna 1210, Austria

## Abstract

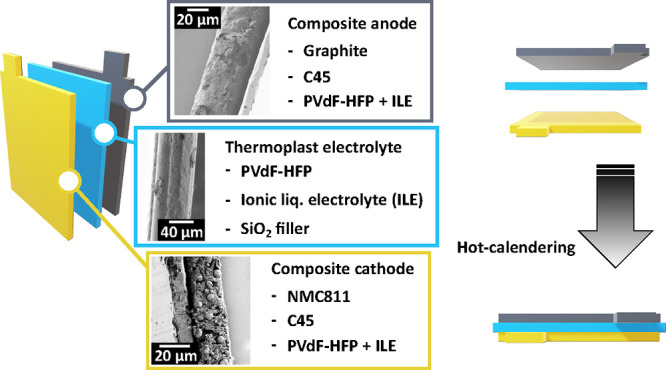

In this work, a battery layup consisting of a poorly flammable ionic liquid electrolyte and a poly(vinylidene fluoride-*co*-hexafluoropropylene) (PVdF-HFP) thermoplastic has been developed along with composite anode and cathode electrodes. The developed gel electrolyte exhibits feasible ionic conductivity of about 1 mS/cm at 30 °C. State-of-the-art active electrode materials, i.e., LiNi_0.8_Mn_0.1_Co_0.1_O_2_ (NMC811) and graphite, have been employed. Full cells were tested in coin and pouch cell format, obtaining capacities of about 120 and 100 mA h/g_NMC811_, respectively, at a C-rate of C/10. Thereby, it was observed that good contact between the individual cell layers is crucial. Recently, it was shown that the mechanical properties of structural batteries, realized by integrating battery cells into carbon fiber-reinforced polymer (CFRP) laminates, depend significantly on the mechanical properties of the cell itself. Hence, to promote the realization of such a structural battery concept, tensile tests were carried out to investigate the mechanical properties of cells as well as the individual components developed in this work. The full cell showed values of 10 GPa and 49 MPa for the Young’s modulus and tensile strength, respectively. Thus, feasible multifunctionality could be verified on the cell level. However, regarding the contributions of the different components, it could be shown that mainly the current collector foils contribute to the mechanical properties, in contrast to the electrode loadings and the gel electrolyte. Additionally, the thermal and chemical stability of the developed system was evaluated, highlighting the importance of these secondary properties for the fabrication of structural batteries, i.e., the integration of cells into load-bearing CFRP laminates. Specifically, it was observed that the developed system is thermally stable up to 150 °C and no HF release was detected upon exposure to ambient conditions.

## 1 Introduction

Multifunctional energy storage or so-called structural batteries^1−4^ have recently attracted lots of interest in academia and industry as conventional energy storage technologies are reaching their theoretical limitations. The promises of using such systems capable of storing energy as well as bearing load are manyfold; however, the expected weight savings and concomitant range extensions for both electric automobiles and aircrafts stand out the most. It was estimated that for a conventional aircraft, range extensions of 11–66% can be achieved by substituting conventional batteries with structural ones.^5^

Different strategies for developing structural batteries have been disclosed, including coaxially coated fibers,^6^ reinforced multilayer stacks,^7,8^ incorporation of cylindrical-,^9^ pouch-,^10^ and thin film batteries^11−13^ into load-bearing structures, as well as using coated carbon fibers as electrodes.^14^ A proper classification of the different approaches according to their degree of multifunctionality is given in refs (1,5,15). Usually, either high electrochemical performance and low mechanical properties or vice versa was achieved, i.e., high degrees of multifunctionality are still lacking.^15^

For conventional lithium-ion batteries, the liquid electrolyte is the main weakness. It does not contribute to the mechanical strength of the battery assembly, and in addition, it prohibits load transfer between the different layers as it does not exhibit any adhesive properties. Therefore, the development of electrolyte layers with balanced electrochemical and mechanical properties limits the development of structural batteries. Two main approaches can be distinguished in this regard. The first one utilizes a biphasic electrolyte (also bicontinuous electrolyte), which combines a mechanically strong polymer (mainly thermosets) with a liquid electrolyte component. Different structures and pore shapes can be envisioned for such electrolytes and have been investigated theoretically.^16^ The other approach exploits the advances in the development of solid electrolytes for use in high-energy-density lithium batteries. Here a monophasic system is implemented as the electrolyte for the structural batteries, which are intrinsically mechanically stable. These electrolytes range from gel electrolytes^6^ to polymer-based ones^17^ as well as ceramic electrolytes.^5^ Depending on the selected material, different mechanical and electrochemical properties can be obtained.

Previous works mainly focused on the electrochemical and mechanical performance of structural batteries, which are the most important regarding the operation of the cells. However, following the approach of the SOLIFLY and MATISSE project (funded by the European Union), i.e., integrating batteries into load-bearing carbon fiber reinforced polymer (CFRP) laminate plates,^58^ also secondary requirements need to be fulfilled. Production facilities of CFRP structures usually operate under ambient conditions. In contrast, battery manufacturing heavily relies on dry room facilities, which enable the handling of otherwise hazardous materials. Especially, the electrolyte components, e.g., LiPF_6_, are a safety concern as they decompose into highly toxic HF when exposed to moisture.^18−22^ Therefore, the assembled structural batteries should comply with the production environment established for load-bearing parts to avoid high costs, which would render this technology economically unattractive. Furthermore, the curing (or consolidation) of CFRP plates relies on elevated temperatures and pressures. Hence, the thermal stability of the structural battery components is very important, as they need to sustain the curing process without impacting the electrochemical and mechanical performance. In addition, it was demonstrated that the mechanical properties of structural batteries, created by integrating battery cells into carbon fiber-reinforced polymer (CFRP) laminates, are strongly influenced by the mechanical characteristics of the cells themselves.^58^ Hence, to advance the development of this structural battery concept, it is necessary to characterize the mechanical properties of the battery cell and its individual components.

For this work, a gel electrolyte was developed which combines poly(vinylidene fluoride-*co*-hexafluoropropylene) (PVdF-HFP) as a thermoplastic together with a SiO_2_ additive providing mechanical properties and a poorly flammable ionic liquid electrolyte, i.e., 3 M lithium bis(fluorosulfonly)imide (LiFSI) in N-propyl-*N*-methylpyrrolidinium bis(fluorosulfonyl)imide (PYR_13_FSI). Similar systems have already been successfully tested for conventional battery applications^23,24^ and the components have been selected to fulfill the needs for application as structural batteries. For providing high tensile modulus, PVDF and derived materials are considered promising due to their high tensile moduli of 0.5–2 GPa.^25−27^ PVdF itself, though, is a complex material (five polymorphs are known^28^ with a high affinity toward crystallization. Preliminary studies have shown a complex interaction with the ionic liquid electrolyte, indicating crystallization effects (see Figure S1 in the SI), thus rendering PVdF not suitable for the manufacturing of gel electrolytes. PVdF-HFP on the other side is known for its more amorphous nature and more beneficial interaction with liquid electrolytes,^29^ although at the cost of the resulting mechanical properties.^30^

Corrosion of current collectors is of major concern for lower-concentrated electrolytes.^31−33^ Hence, a rather high concentration of 3 M of LiFSI in PYR13FSI was chosen for this work, since it was shown that higher concentrated electrolytes can suppress corrosion phenomena.^34^ In addition, PYR13FSI exhibits good compatibility with both NMC and graphite,^23,35^ which were used as cathode and anode active material in this work, respectively, as compared to, e.g., imidazolium-based systems. Furthermore, the Li salt LiFSI is considered to yield a more beneficial SEI layer compared to the more commonly used LiTFSI salt.^36^

Gel electrolytes of this kind are promising for structural batteries with regard to their balanced properties. A liquid electrolyte + separator system might have a similar tensile modulus compared to gel electrolytes (in the MPa range); however, it is unable to transfer loads efficiently due to the liquid interfaces.^37^ The brittle nature of ceramic solid electrolytes, especially oxides, and the lack of coherent interfaces between the ceramic electrolyte and the electrodes render them not suitable for structural battery application. Furthermore, polymer electrolytes without the addition of a liquid component as plasticizers suffer from poor ionic conductivities.^38^ Therefore, a gel electrolyte incorporating a poorly flammable ionic liquid is considered to be a feasible option taking into account other important parameters like safety, processability, and reliability. In addition, the gel electrolyte must be incorporated into the electrodes to form proper ionic conduction pathways. Compared with the use of gels in conventional batteries, adhesion between all layers is of utmost importance for structural batteries to enable efficient load transfer. Liquids at the interfaces, however, inhibit the transfer of loads and thus result in poor mechanical properties. Therefore, the wetting of the electrodes with a liquid electrolyte, as applied in conventional battery applications, is unfavorable for structural batteries.

In most reports on structural batteries,^6,8,14,39,40^ LiFePO_4_ (LFP) is used as cathode material, as it offers a highly stable platform to test different electrolyte materials. Nevertheless, they lack the energy density required to compete with state-of-the-art batteries using liquid electrolytes. Therefore, in this work the developed gel electrolyte is combined with a composite LiNi_0.8_Mn_0.1_Co_0.1_O_2_ (NMC811) cathode and composite graphite anode to form a full structural battery cell. To the best of the authors’ knowledge, a system consisting of such a PVdF-based gel polymer electrolyte together with composite electrodes containing this electrolyte has not been reported yet.

The electrochemical performance was determined by means of electrochemical impedance spectroscopy (EIS) of the developed gel polymer electrolyte and by using galvanostatic cycling with potential limitations (GCPL) for half as well as full-cell assemblies. The mechanical properties of individual components, as well as full cells, have been analyzed by tensile and peel tests. Furthermore, secondary requirements such as the chemical and thermal stabilities of the developed components have also been evaluated. Thereby, it was shown that the presented cell chemistry is promising for integration into CFRP laminate plates and thus may pave the way for multifunctional energy storage. However, it is also outlined where this approach needs improvement, indicating how some of these points may be addressed.

## 2 Experimental Section

### 2.1 Preparation of Ionic Liquid Electrolyte

All preparation steps have been performed in a dry room (dew point about −50 °C) unless otherwise stated. First, the ionic liquid electrolyte (ILE) was prepared by dissolving appropriate amounts of Li-salt in the ionic liquid to obtain a 3 M solution. An aluminum bottle was used as a container, and a polytetrafluoroethylene (PTFE) covered stirring bar in combination with a magnetic stirrer was used for mixing. The lithium salt and ionic liquid were purchased from Solvionic and used as received. The selected materials were lithium bis(fluorosulfonly)imide (LiFSI, 99.9%, H_2_O: 20 ppm max., Solvionic) and N-propyl-*N*-methylpyrrolidinium bis(fluorosulfonyl)imide (PYR_13_FSI, 99.9%, Solvionic). After the addition of the salt to the ionic liquid, the solution was stirred for at least 16 h to ensure complete dissolution.

### 2.2 Preparation of Thermoplast-Based Gel Electrolyte

SiO_2_ nanoparticles (nanopowder, 10–20 nm particle size (BET), 99.5% trace metals basis, Sigma-Aldrich) were dispersed in 1-methyl-2-pyrrolidone (NMP, ≥ 99.8%, Carl Roth) using a dissolver (IKA Eurostar 60) and a dispersion blade. High shear rates were applied for 30 min by setting the rpm values to 500, thus ensuring proper dispersion of the SiO_2_ particles. Then, poly(vinylidene fluoride-*co*-hexafluoropropylene) (PVdF-HFP, Sigma-Aldrich, avg *M*_n_ about 400k) was added and dissolved into the SiO_2_ dispersion using an anchor type stirring head and low rpm values of around 100 rpm for 16 h. Finally, the ionic liquid electrolyte was added, and the mixture was stirred for another 2 h at low rpm values. A materials ratio of SiO_2_:PVdF-HFP:ILE of 7:26:67 (vol./vol.) = 11:28:61 (wt./wt.) and a solid content of 34 wt % (for which the ILE is also considered as solid as it will remain in the electrolyte after drying) of the slurry used during processing were utilized. The thus-prepared slurry was tape cast onto a glass plate using a doctor blade with a 500 μm gap size. The cast film was further dried under vacuum at 80 °C for 3 days. After drying, the electrolyte film can be easily peeled from the glass plate, resulting in a freestanding film with a thickness of around 120 μm. Possible pores and trapped air bubbles are removed by hot-calendering of the film at 122 °C and 6 mm/s, reaching final thicknesses of 30–40 μm. During calendering, the electrolyte is sandwiched between two polyimide foils to avoid any contamination of the calender with the ILE. The final electrolyte is a colorless transparent film and is stored between two polypropylene foils until further use.

### 2.3 Preparation of Composite Electrodes

For the composite electrodes, LiNi_0.8_Mn_0.1_Co_0.1_O_2_ (HED NCM 811, BASF) and graphite (carbon microbeads AZBH4, NCK) were used as active materials (AM). First, the conductive additive (C45, Imerys) was dispersed in NMP using a dissolver and dispersion blade. The dispersion was mixed at 500 rpm for 30 min. Then the active material was added, and the stirring process continued for 1 h. In the next step, the PVdF-HFP binder was added and an anchor-type stirring head was used to dissolve the polymer at 100 rpm for 16 h. Finally, the ILE was added and the slurry was mixed for another 2 h. The AM:C45:PVdF-HFP:ILE ratios for the cathode and anode were 68:5:7:20 (wt./wt.) = 41:9:12:38 (vol./vol.) and 45:6:11:38 (wt./wt.) = 49:5:9:37 (vol./vol.) using solid contents of 62 and 46 wt %, respectively.

The prepared slurries were tape cast onto carbon-coated Al-foil (TianJin Haiiwei Technology Co., Ltd.; 16 μm total thickness: 15 μm Al + 1 μm carbon coating) for the cathode or onto surface-treated Cu-foil for the anode (PHC Cu foil SE-Cu58, Schlenk; 10 μm total thickness). The doctor blade gap size was set to 120 and 200 μm for the cathode and anode coatings, respectively. A cathode loading of around 1 mA h/cm^2^ (around 5 mg/cm^2^) was targeted, considering the maximum specific capacity of 190 mA h/g for NMC811.^53^ The corresponding anode-specific charge was selected to be 1.5 mA h/cm^2^ (with 372 mA h/g being the maximum specific capacity of graphite, i.e., LiC_6_) resulting in an N/P ratio of 1.5. After the casting procedure, the electrodes were dried at 80 °C under vacuum for 3 days. The dried electrode sheets were further hot-calendered at 122 °C for densification and to remove most of the pores formed by evaporation of the solvent.

### 2.4 Cell assembly and Electrochemical Testing

For the preparation of single-layer pouch cells, composite cathodes and anodes were cut into rectangular shapes with rounded edges of 68 × 98 mm^2^ and 70 × 100 mm^2^ using a semiautomatic die cutter. The electrolyte sheets were cut manually using a scalpel into rectangular shapes of 72 × 102 mm^2^. Cell stacks were manually prepared by first placing the anode on top of the electrolyte sheet and subsequently placing the cathode on the opposite side of the electrolyte. The thickness of the assembled cell was measured (usually around 160–170 μm), and for some cells, the stack was hot-calendered at 100 °C with a gap of 140–150 μm (20 μm less than the measured thickness) to ensure proper contact between all layers. Then, Al (for the cathode) and Ni (for the anode) tabs are ultrasonically welded onto the two electrodes, and the cell is finally sealed in a pouch foil (Dai Nippon Printing, D-EL408PH(3)S-250) under reduced pressure. The entire preparation process of pouch cells was done in a dry room.

Coin cells in CR2016 format were fabricated in a glovebox (O_2_ and H_2_O content of less than 0.1 ppm) by cutting composite electrodes and gel electrolytes into disks with a diameter of 15 and 18 mm, respectively. Cells were assembled by stacking the anode, electrolyte, and cathode in the smaller part of the CR2016 casing. Furthermore, a stainless steel disk-shaped spacer (15 mm diameter, 0.5 mm thickness) and a stainless steel wave spring (15 mm diameter, 1.1 mm thickness) were used. In order to seal the cell, a pressure of 7 MPa (1000 psi) was applied for 10 s. A similar procedure was used for the fabrication of half cells; however, a Li chip was used instead of one of the composite electrodes. Prior to the assembly, the Li chips were cleaned by scratching off the surface layers using a scalpel. The Li chips were further rolled in between two pouch foils with a glass cylinder, thus obtaining cleaned and smooth surfaces. For some of the half-cell tests, composite electrodes were wetted with 20 μL of the same ILE that was used for the preparation of the gel electrolyte. For determining the ionic conductivity of the gel electrolyte, a disc of 18 mm diameter was cut from the electrolyte film and placed inside an ECC-Std. (EL-Cell) setup. The cell impedance was measured using a BIOLOGIC-VSP potentiostat in a frequency range of 1 MHz to 1 Hz. The thickness of the electrolyte was determined prior to the assembly. An excitation potential of 10 mV was applied and measurements were conducted in potentiostatic mode (PEIS) between 0 and 80 °C. Electrochemical cycling of the cells was done at 25 °C using galvanostatic cycling with potential limitations (GCPL) between 3.0 V and a maximum of 4.3 V. For rate capability tests, different current densities corresponding to C-rates of C/10, C/5, C/2, and 1C were applied.

### 2.5 Physico-Chemical and Mechanical Testing

Differential scanning calorimetry (DSC) measurements were conducted by using a TA Discovery DSC instrument (TA Instrument). The measurements were conducted under a N_2_ atmosphere at temperatures between −50 and 200 °C. A heating rate of 10 °C/min was applied to all samples. Film samples were first cut into approximately 2 mm × 2 mm shapes and put into standard aluminum pans with lids as sample containers.

Fourier-transform infrared spectroscopy (FTIR) measurements were conducted by using a Spectrum Two instrument (PerkinElmer). A wavenumber range of 4000 to 400 cm^–1^ was selected. A total of four scans were recorded for each sample using a resolution of 1 cm^–1^. Thermogravimetric analysis (TGA)/FTIR measurements were conducted using a NETSCH STA 449 F1 and a Bruker INVENIO-S with a TGA cell. The measurements were conducted under a synthetic air atmosphere (80% N_2_; 20% O_2_) between 40 and 150 °C. A heating rate of 5 °C/min was applied for all samples. The measured samples were placed into alumina crucibles. The FTIR instrument measured the absorbance of the evolved gases in the range of 600 to 4400 cm^–1^.

SEM micrographs were recorded on a ZEISS Supra 40 electron microscope. A 2 kV acceleration voltage was applied. All samples were mounted on sample holders using Ag paste inside a dry room. Cross sections were manually prepared using a scalpel. To avoid exposure to ambient conditions during the transfer of the samples to the instrument, they were put into a sealed container, which was opened only right before shuttling them inside the instrument.

The tensile properties of polymer electrolytes, composite electrodes, and battery cells were determined using a universal dual-column mechanical testing frame (Instron 5969, Instron) equipped with a 1 kN load cell. The specimens were cut into dog bone shapes with a neck width of 2 mm and a total length of 30 mm (1BB in BS EN ISO 527); the gauge length was marked to be 10 mm in the center of the specimens. The specimen thickness was determined using a Sumet MB-P-20. The clamping distance was 20 mm. The electrolyte was tested at a speed of 10 mm/min, while the electrode and battery cell were tested at a speed of 1 mm/min. The strain was determined using a video extensometer (IMT-CAM018, IMETRUM).

Hydrofluoric acid release was monitored using a Dräger X-am 5100 HF/HCl sensor with a measurement range between 0 and 30 ppm of HF/HCl (0.1 ppm resolution) in a conventional desiccator. Samples were placed together with the HF sensor into the desiccator under ambient atmosphere (25°, 45–55% relative humidity) and subsequently closed. Then HF formation was monitored by checking the values on the HF sensor after different time steps.

Adhesion strength of the cell components were tested using a 180° peel tester (EKM-5KN, Jinan Marxtest Technology Co., Ltd.). Samples were cut into 20 × 30 mm^2^ shapes and glued onto a steel sample holder using double-sided duct tape. A peeling speed of 20 mm/min was applied for all samples.

## 3 Results and Discussion

### 3.1 Physico-Chemical and Electrochemical Characterization of Thermoplast-based Gel Electrolyte Film

**Figure 1:**
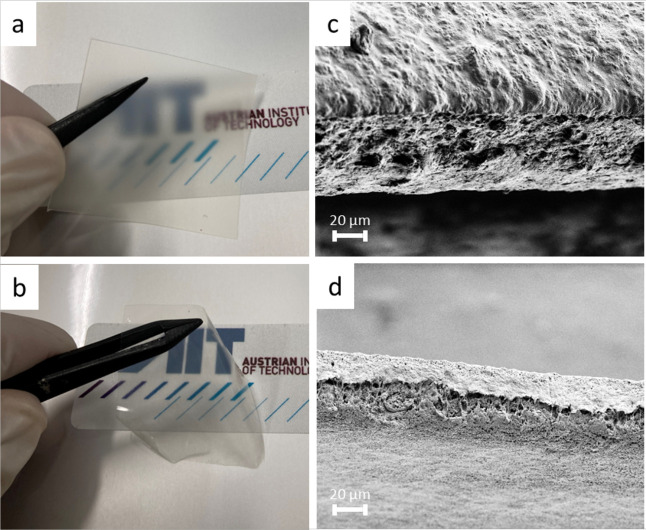
Photographs (a, b) and SEM micrographs (c, d) of the electrolyte films before (a, c) and after hot calendering (b, d).

After the cast electrolyte films were dried, opaque freestanding films were obtained without any visible liquid component at the surface. SEM analysis showed that a rough surface formed after evaporation of NMP. After hot-calendering, though, a clear film was obtained, showing a much smoother surface (cf. Figure 1a–d). In addition, the thickness was reduced to 30–40 μm, which is close to that of separators used in conventional batteries. Such a calendering of gel electrolytes has been reported to increase ionic conductivities.^41^

The densities of the films before and after hot-calendering were determined by cutting 18 mm diameter discs from the electrolyte films and recording their masses. From the geometric volume of the films (area × thickness) and the respective mass, the apparent densities could be determined. The as-cast samples showed lower densities of 1.38 g/cm^3^ compared to the calendered ones, which showed values of 1.52 g/cm^3^, close to the one obtained from the rule of mixtures of the components, i.e., 1.6 g/cm^3^. Thus, it can be assumed that the porosity (caused by entrapped air) could be decreased significantly by the calendering step.

**Figure 2:**
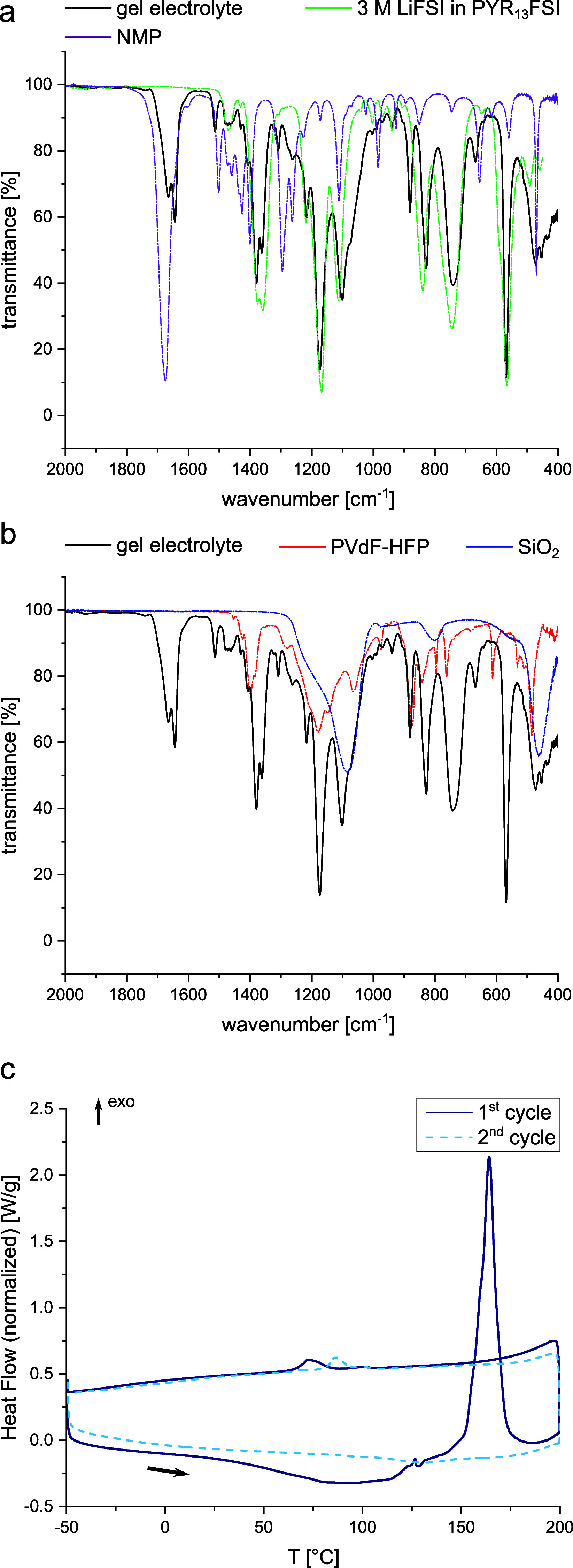
(a, b) FTIR spectra of the gel electrolyte as well as of its components, i.e., SiO2, PVdF-HFP, 3 M LiFSI in PYR13FSI, and NMP (solvent). (c) DSC thermogram of the gel electrolyte from −50 to 200 °C.

The FTIR spectra of the electrolyte as well as of the pristine components are depicted in Figure 2a,b. The absorption bands for the components SiO_2_, PVdF-HFP, and the ionic liquid electrolyte (3 M solution of LiFSI in PYR_13_FSI) can be readily found in the spectra of the gel electrolyte films. Furthermore, additional bands can be observed, with the most pronounced being at wavenumbers of 1665 and 1644 cm^–1^. These cannot be related to any of the precursors and thus seem to be characteristic of the gel electrolyte.

It has been reported previously that solvents used for the processing of PVdF-based materials can be entrapped within the polymer host and can lead to additional FTIR bands. The solvents act as plasticizers and cannot be removed even after prolonged drying.^42,43^ However, the entrapped solvents also seem to increase the ionic conductivity of the electrolytes and thus can improve the electrochemical performance. In Figure 2a, the FTIR spectra of NMP and the gel electrolyte can be readily compared. Indeed, some of the additional bands can be explained by retained solvent; however, some bands seem to be shifted to higher wavenumbers, indicating a different chemical environment of the entrapped solvent molecules compared to the pristine solvent. In addition, the band at 1644 cm^–1^ does not seem to be related to entrapped solvent molecules. Interactions of the Li-salt of the ionic liquid electrolyte and the polymer matrix also need to be considered in this regard. For polymer electrolytes without liquid components, the complexation of LiTFSI in a PVdF-HFP matrix was reported to result in an additional band in the FTIR spectra at 1640 cm^–1^.^44^ Similar interactions of the polymer matrix with the LiFSI salt stemming from the ionic liquid electrolyte are assumed for the gel electrolyte prepared in this work. Diffusion of the Li-salt from the ionic liquid into the polymer matrix is presumed to result in the observed spectra for the gel electrolyte. Especially at elevated temperatures, i.e., during the drying step, the Li-salt may interact with the PVdF-HFP chains.

The DSC thermograms of the prepared gel electrolyte are depicted in Figure 2c. A broad endothermic peak starting from around 50 °C and ending at 125 °C may actually result from the merging of peaks. One peak might be attributed to the melting range of the material. The pristine PVdF-HFP thermoplastic shows a melting range of 140–145 °C according to the specifications of the manufacturer. After processing of the gel electrolyte, however, the melting range seems to be shifted to lower temperatures. LiFSI salt was reported to have a melting point between 124 and 145 °C.^50,55,56^ Residual solvent molecules, as well as interactions between the ionic liquid electrolyte and the polymer matrix, may be responsible for the shift of the melting range to lower temperatures. Some impurities may be responsible for the onset of the endothermic peak at around 50 °C, which is visible only in the first heating cycle. At 150 °C, a sharp exothermic peak arises in the thermograph, which is only visible for the first heating sequence and does not appear in subsequent heating steps. Although LiFSI is generally considered a safe and highly stable alternative to conventionally used Li-salts like LiPF_6_, some reports indicated limited thermal stability, especially when traces of moisture are present. Huang and Hollenkamp^45^ reported that depending on the moisture content of LiFSI, the thermal stability of the salt changes from 183 °C to only 120 °C. A highly exothermic decomposition reaction accompanied by the formation of Li_2_O was assumed. Similar findings were reported by Kerner et al.^46^ who analyzed and compared commercial LiFSI sources. The highly exothermic behavior at elevated temperatures also led to reconsiderations regarding the safety evaluation of these electrolytes.^47,48^ We prepared the gel electrolyte films in a dry room, and thus minor impurities of moisture cannot be avoided. This seems to be responsible for the limited thermal stability of the electrolyte films observed during the DSC measurements.

**Figure 3:**
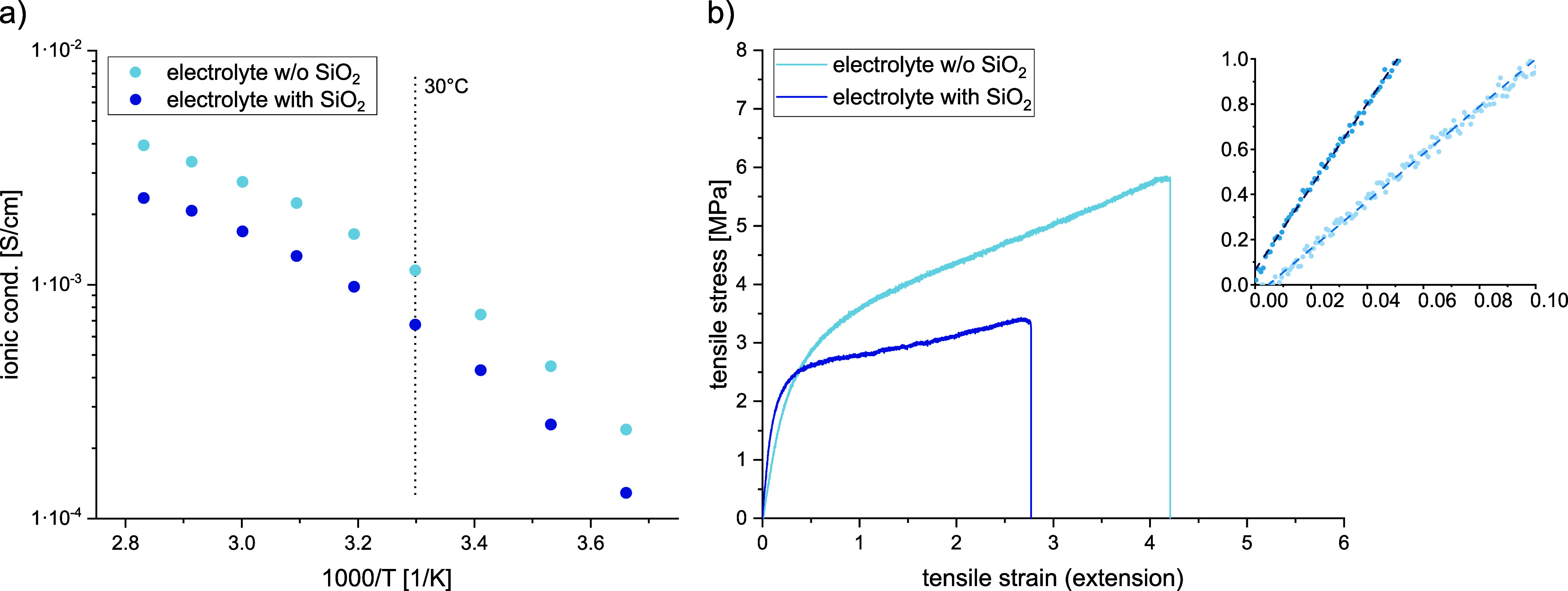
(a) Ionic conductivities and (b) tensile stress of gel electrolytes with and without SiO_2_ filler. The inset in (b) shows a magnification of the linear domain of the tensile test.

The electrolyte layer was further characterized by using PEIS to determine the ionic conductivity. The cell containing the electrolyte layer between two ion-blocking stainless steel electrodes was put inside a climate chamber and PEIS measurements were conducted at temperatures between 0 and 80 °C. Exemplary impedance spectra and corresponding fits are shown in Figure S11 in the SI. During the initial heating of the sample, the contact between the electrolyte and the electrodes usually improves. Therefore, the ionic conductivities have been evaluated only during the cooling of the samples, as presented in Figure 3a. The ionic conductivity of the electrolyte film with and without SiO_2_ filler at 30 °C amounts to 7 × 10^–4^ and 1.1 × 10^–3^ S/cm, respectively.

Tensile testing of the electrolyte was conducted to enable a first evaluation of the multifunctionality of the prepared electrolyte. The electrolytes with and without SiO_2_ filler were tested and the results are depicted in Figure 3b. Without any filler material, the electrolyte films possessed Young’s moduli of 10.5 ± 0.02 MPa. The addition of the silica filler, though, increased the Young’s modulus to 18.4 ± 0.1 MPa, indicating the potential of filler materials to increase the modulus of gel electrolytes. The tensile strength was determined as well and amounted to 4.9 ± 0.5 and 3.5 ± 0.3 MPa for the gel electrolyte with and without SiO_2_ filler, respectively. Thus, the filler material could increase the modulus, however, it decreased the ultimate tensile strength.

### 3.2 Physico-Chemical Characterization of Composite Electrodes

**Figure 4:**
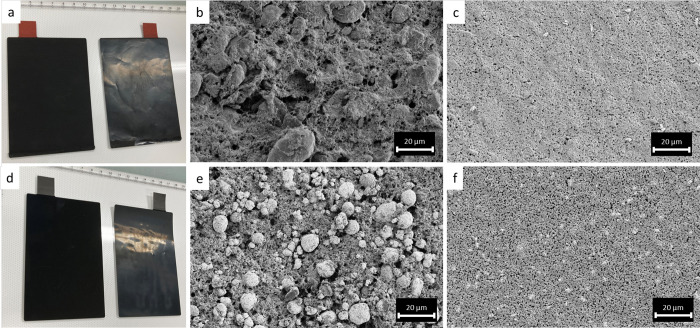
Photographs of composite anode (a) and composite cathode (d). SEM images of the composite anode (b, c) and the composite cathode (e, f) before (b and e, respectively) and after hot-calendering (c and f, respectively).

The composite electrodes possessed rough surfaces after drying, whereas smooth and shiny surfaces were obtained after hot-calendering (Figure 4). In the SEM micrographs, the spherical active material particles for both the composite anode and cathode can be readily observed. Also, the porosity seems to be rather high and gaps between the active material and the gel electrolyte are visible.

After calendering, though, a smooth surface is observed, and no bare active material particles can be seen anymore. The cross sections of the electrodes are shown in Figure S2a–d in the Supporting Information (SI) and show the effect of the calendering step on the electrode morphology. The as-cast electrodes comprise a loose network of active material particles held together by the gel electrolyte. After calendering, a dense layer is observed for both electrodes, and the active material particles are tightly enclosed by the gel electrolyte.

Similar to the density of the electrolyte, the apparent density of the electrodes was measured before and after the hot-calendering step. As depicted in Figure S2e in the SI, the densities increased after the calendering. For the composite anodes, as-cast electrodes are densified from 1.16 to 1.32 g/cm^3^, quite close to the theoretical value of 1.45 g/cm^3^ obtained from the rule of mixtures of all components. For the composite cathodes, though, a lower degree of densification is obtained, i.e., the electrodes are densified from 1.18 to 1.53 g/cm^3^, which is still far from the theoretical value of 2.92 g/cm^3^.

**Figure 5:**
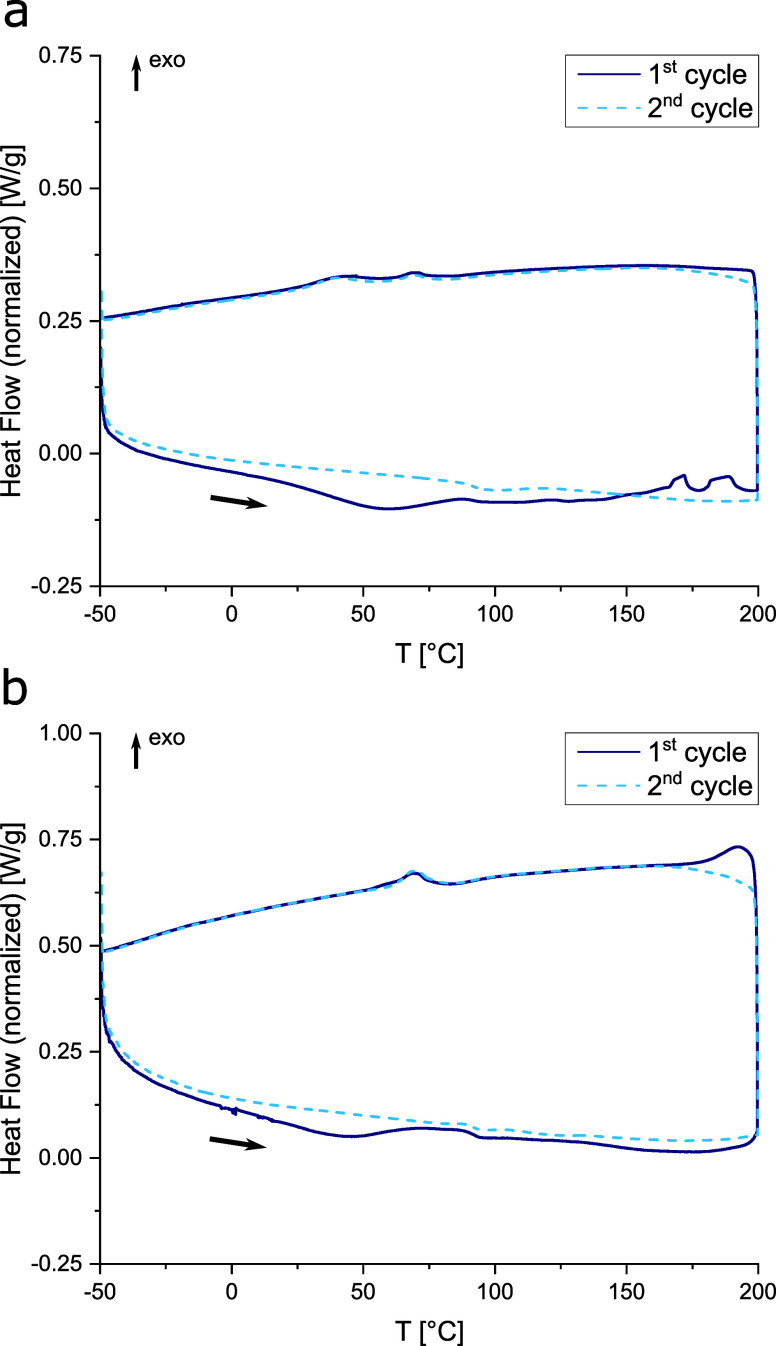
DSC thermographs of a) the composite anode and b) the composite cathode from −50 to 200 °C.

The DSC thermograms of the composite anode and cathode are depicted in Figure 5. During the first heating step, two endothermic peaks could be observed at about 50 and 100 °C. The latter is within the melting range of the gel electrolyte as already discussed for Figure 2c. The former, though, seems to stem from some impurities, as it only occurs during the first heating step. In the second cycle, this peak cannot be observed for both electrodes. The exothermic peak at 150 °C, which shows the decomposition of the gel electrolyte, could not be observed in the case of the composite electrodes. Only some minor peaks at 165 and 185 °C were recorded in the case of the anode, whereas an exothermic signal at 200 °C was observed for the cathode. Thus, slightly higher thermal stability could be achieved compared to that of the electrolyte. The SiO_2_ filler particles, absent in the composite electrodes, are susceptible to H_2_O adsorption,^61^ which can catalyze decomposition reactions in the gel electrolyte,^45^ thereby reducing its thermal stability compared to that of the electrodes.

#### 3.2.1 Chemical Stability of Cells, Cell Components, and Precursors

Fluorinated compounds such as LiPF_6_ used in conventional batteries are known to release toxic HF during reactions with even trace amounts of H_2_O.^18−22^ Moreover, it was shown that H_2_O impurities in such batteries lead to a significant capacity decrease.^57^ Therefore, these materials can only be handled in glovebox or dry-room conditions. However, the assembly of load-bearing composite fiber-reinforced polymer (CFRP) laminates usually takes place under ambient conditions. Thus, the integration of structural batteries into CFRP laminates poses risks regarding safety and performance if the materials are not properly selected. Hence, to enable the application of structural batteries, the selected components should withstand exposure to ambient conditions for the time it takes to assemble and incorporate them into CFRP laminates. In this work, the developed electrolyte contains fluorinated polymers and ionic liquids as well as lithium salts. As such, the potential threat of HF release is imminent and needs to be addressed properly. The FSI^–^ anion used for the electrolyte is reported to release only insignificant amounts of HF in the low ppm range upon exposure to moisture^49,50^ and thus is considered to be a safe component. Nevertheless, all components, i.e., the ionic liquid electrolyte, the gel electrolyte, the composite cathode, and the composite anode, have been tested for HF release using a commercial HF detector (measurement range between 0 and 30 ppm with a resolution of 0.1 ppm). The samples were sealed under ambient conditions in a desiccator together with the detector, and the HF values were recorded at different times. The measurement setup is shown in Figure S3 and the results are summarized in Table S1 (see SI). Clearly, no HF release was observed for any of the developed battery components. Furthermore, the used ionic liquid electrolyte with a high concentration of the LiFSI salt also did not show any HF release, even after 24 h. Thus, it can be assumed that the developed cell components are safe to handle under ambient air for integration into CFRP laminates.

**Figure 6:**
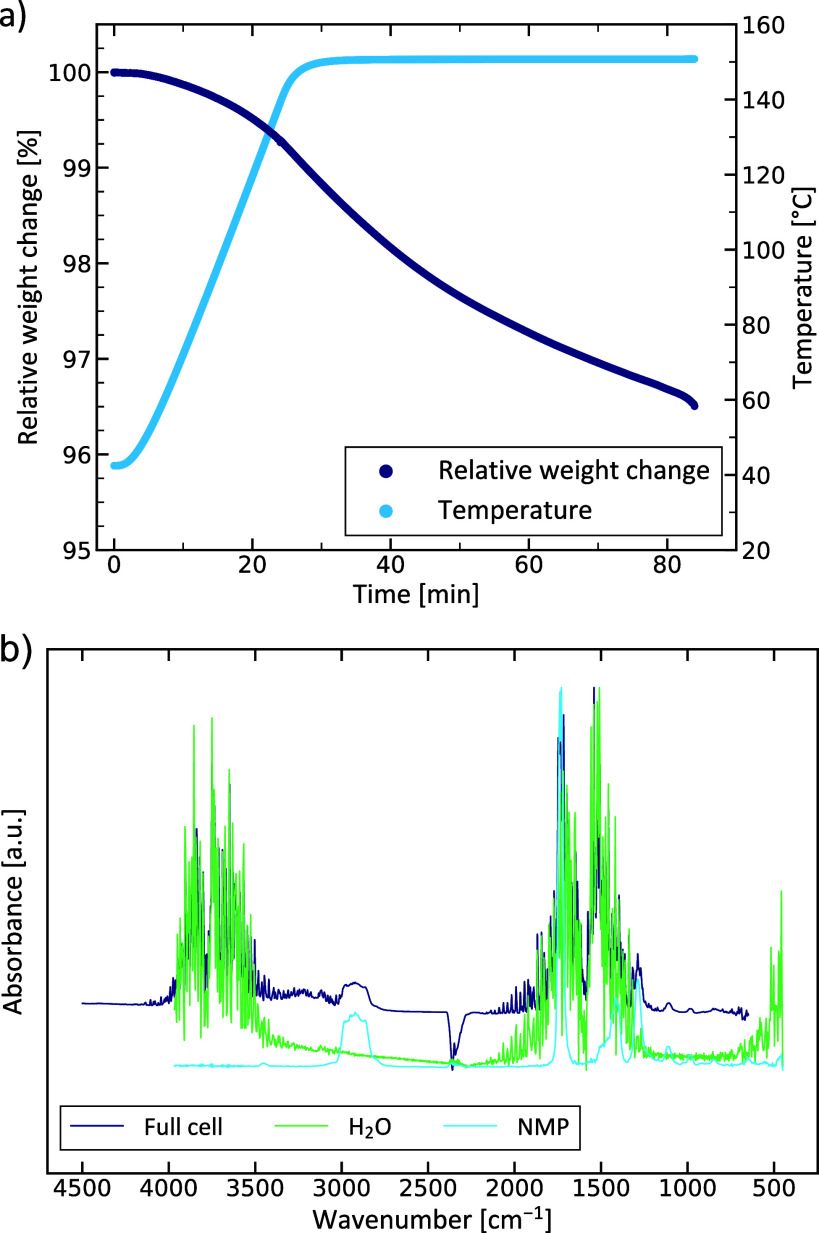
TGA measurement (a) of a full cell and corresponding FTIR spectrum (b) recorded at 150 °C (after about 32 min). The data of the H_2_O and NMP spectra are taken from ref (51).

In addition, TGA/FTIR measurements of full cells were conducted in order to analyze any decomposition reaction and/or gas release under elevated temperatures. The corresponding results are depictedin Figure 6. The relative weight decreases over the course of the measurement to about 96.5% with most of the weight loss occurring at temperatures above 140 °C. The shown FTIR spectrum was recorded after 32 min, i.e., at 150 °C, which is associated with the maximum of the IR detection. By comparing the spectrum of the full cell to reference spectra from databases,^51^ it can be concluded that the evolved gases and thus the weight loss may be ascribed to the evaporation of H_2_O and NMP. Hence, some moisture and solvent seem to be entrapped in the cells after manufacturing in alignment with the DSC and FTIR results of the electrolyte (see above). Moreover, a comparison with FTIR data of HF^52^ indicates that no toxic HF was released even under elevated temperatures.

### 3.3 Electrochemical Performance of Cell Assemblies

**Figure 7:**
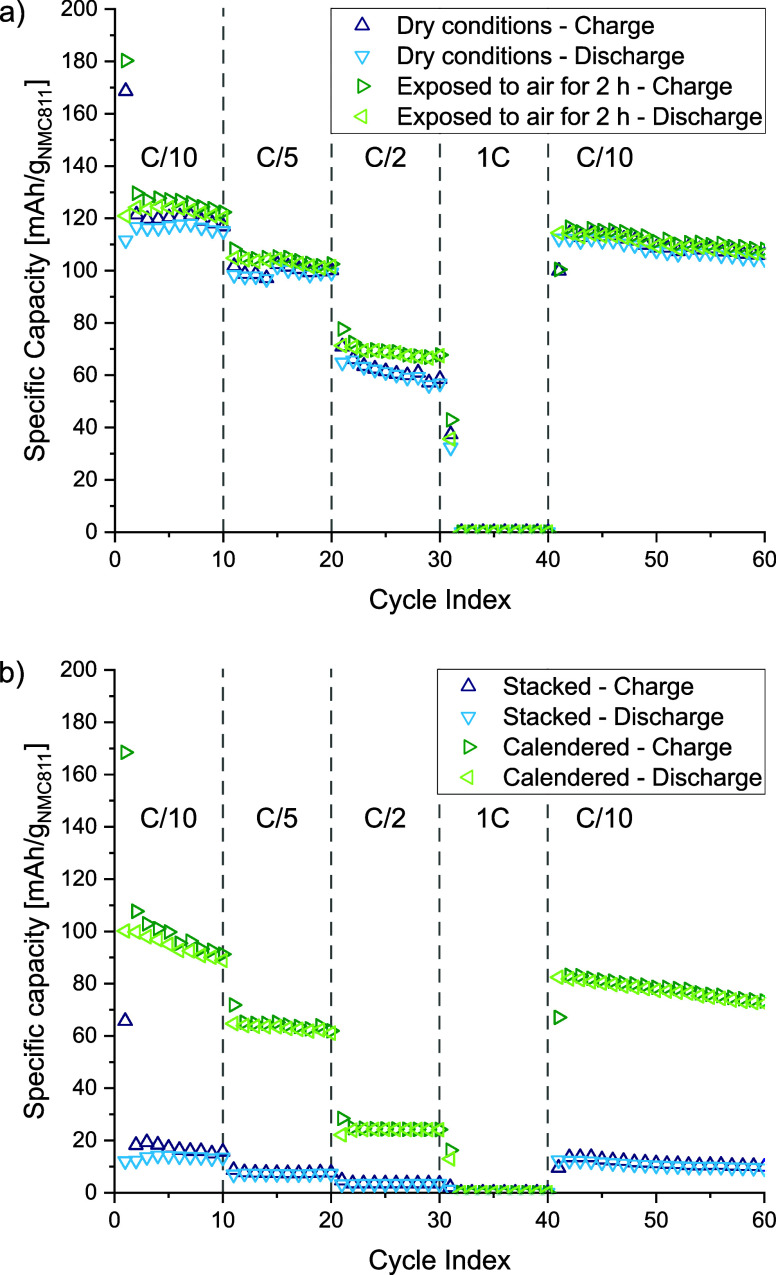
Specific capacity of (a) full cells in coin cell format at different C-rates assembled under dry conditions as well as after exposure to ambient air. (b) Full cells in pouch cell format with and without a final hot-calendering step, respectively.

Full cell assemblies in coin as well as pouch cell format were tested by galvanostatic cycling with potential limitations (GCPL) using a potential window of 3.0–4.2 V (vs Li/Li^+^). Different C-rates have been applied to evaluate the rate-capability of the cell. Figure 7a depicts the results of coin cells observing capacities of about 120 mA h/g_NMC811_ at a C-rate of C/10. The cells could be cycled up to C-rates of C/2 with some capacity fading over time. Remarkably, similar capacity values were obtained with cells whose components were exposed to ambient air for 2 h prior to the assembly. Hence, together with the results showing no HF release even after 24 h, we conclude that the developed cell chemistry seems to be viable for handling under ambient conditions, e.g., for the integration into load-bearing CFRP laminates.

The results obtained from pouch cell tests are depicted in Figure 7b. For cells whose individual layers were only stacked on top of each other during assembly, rather poor performance was achieved. Even at low current densities, i.e., with a C-rate of C/10, only 12 mA h/g_NMC811_ were obtained. Poor contact between the composite electrode layers and the gel electrolyte was assumed to be the main reason for this observation, as air bubbles between the layers could be seen during assembly. Therefore, an additional hot-calendering step was applied after cell assembly to improve the contact between all layers and remove trapped air bubbles as much as possible. The electrochemical performance of postcalendered pouch cells is depicted in Figure 7b.

The hot-calendering of the assembled cell led to a significant performance improvement. In this case, specific charge values of 100 mA h/g_NMC811_ could be achieved at C/10 and cycling was possible up to high C-rates of C/2. These results clearly indicate the importance of good contact between the individual cell layers. The better performance in coin cells may also be attributed to better contact between layers since it results from the pressure applied during sealing. The corresponding potential profiles and Coulombic efficiencies are shown in Figures S4–S6 (SI).

**Figure 8:**
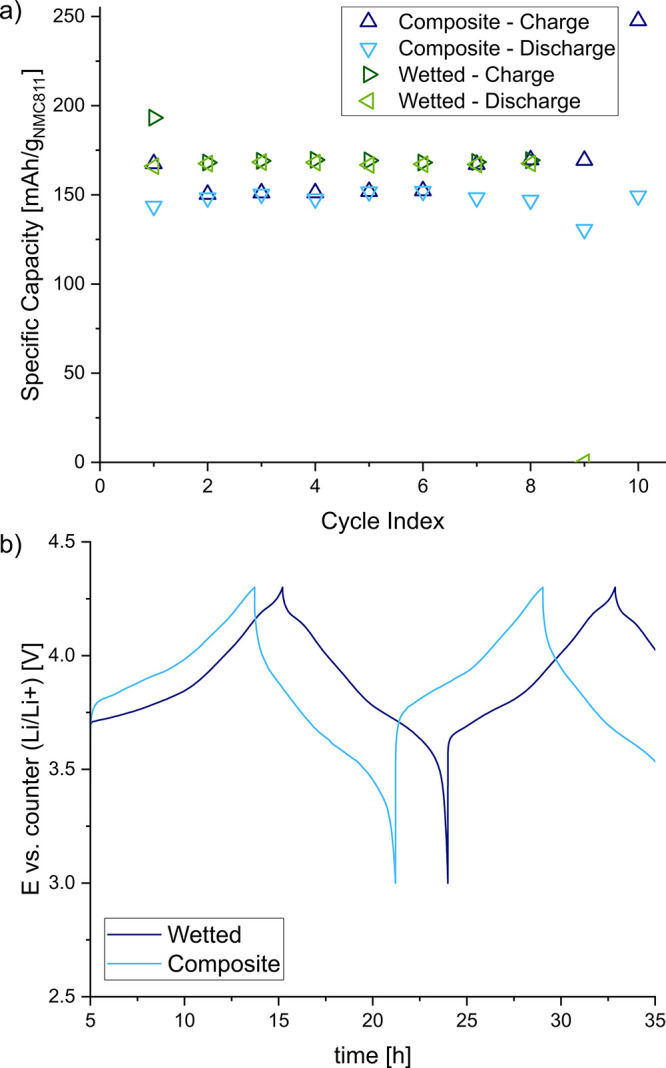
Specific capacities (a) and potential profiles (b) of NMC811 half cells in coin cell format with (denoted as wetted) and without (denoted as composite) an additional 20 μL of ILE.

**Figure 9:**
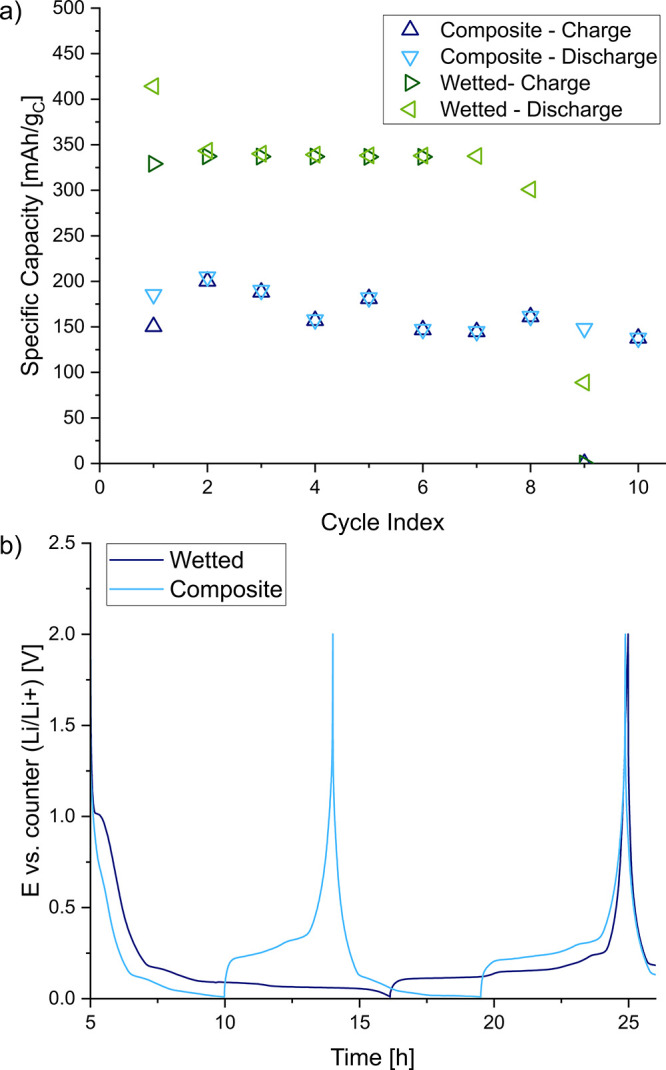
Specific capacities (a) and potential profiles (b) of graphite half cells in coin cell format with (denoted as wetted) and without (denoted as composite) an additional 20 μL of ILE.

However, the theoretical capacity of NMC811 amounting to 190 mA h/g_NMC811_^53^ was not reached. The irreversible capacity loss of the first cycle indicates loss of lithium during the formation of an SEI layer at the anode interface and potentially a CEI layer on the cathode side. In order to gain further insights, half-cell measurements of the composite electrodes in coin cell format with a Li chip counter electrode were conducted. Galvanostatic cycling of half cells at a C-rate of C/10 between 3 and 4.3 V of the NMC811 composite cathode reveal capacities of around 150 mA h/g_NMC811_ (see Figure 8a). Interestingly, higher capacity values of around 170 mA h/g_NMC811_ are obtained when 20 μL of the ILE were added on top of the composite NMC811 cathode during the assembly (denoted as wetted). This difference is even larger in the case of galvanostatic cycling composite graphite anode half cells at a C-rate of C/10 between 0 and 2 V: between 150 and 200 mA h/g_C_ were achieved with the bare composite anode compared to around 340 mA h/g_C_ when 20 μL of the ILE were added. Thus, wetting with 20 μL of the ionic liquid electrolyte leads to capacity values that are closer to the theoretical limits (190 mA h/g_NMC811_ for NMC 811^53^ and 372 mA h/g_C_ for graphite, i.e., LiC_6_). From the voltage profiles (Figures 8b and 9b), it is clearly visible that the bare composite electrodes have higher overpotentials compared to the wetted ones. Some instabilities observed in the half-cell tests after a few cycles may be attributed to the instability of the gel electrolyte against the Li metal counter electrode.

The wetting of the composite electrodes may have several impacts. First, it can be assumed that the ionic conductivity of the composite electrodes increases simply due to the higher incorporated amount of ILE. Moreover, the additional ILE may also affect the SEI and CEI formation. In general, it seems that the additional ILE has a greater effect at the anode side since the wetting led to a higher capacity increase compared to the cathode side. A more rigorous work to further elucidate the causes of these phenomena and to improve the electrochemical performance is planned but would exceed the scope of this work.

In the case of pouch cells (without adding ILE to the composite electrodes during assembly), an energy density of 370 W h/kg_NMC811_ could be achieved considering an average discharge potential of 3.7 V. Addition of the anode active material and the passive components, i.e., current collectors, gel electrolyte, additional components of the electrodes (conductive additive, electrolyte) to this calculation, though, reduces the energy density to only 48 W h/kg_Full Cell_. The main contribution to the weight can be assigned to the current collectors, which contribute to 43% (cf. Figure S7 in the SI). Hence, current collectors as passive components reduce the energy density significantly.

### 3.4 Mechanical Performance of Cell Assembly (Tensile Test and Peel Test)

**Figure 10:**
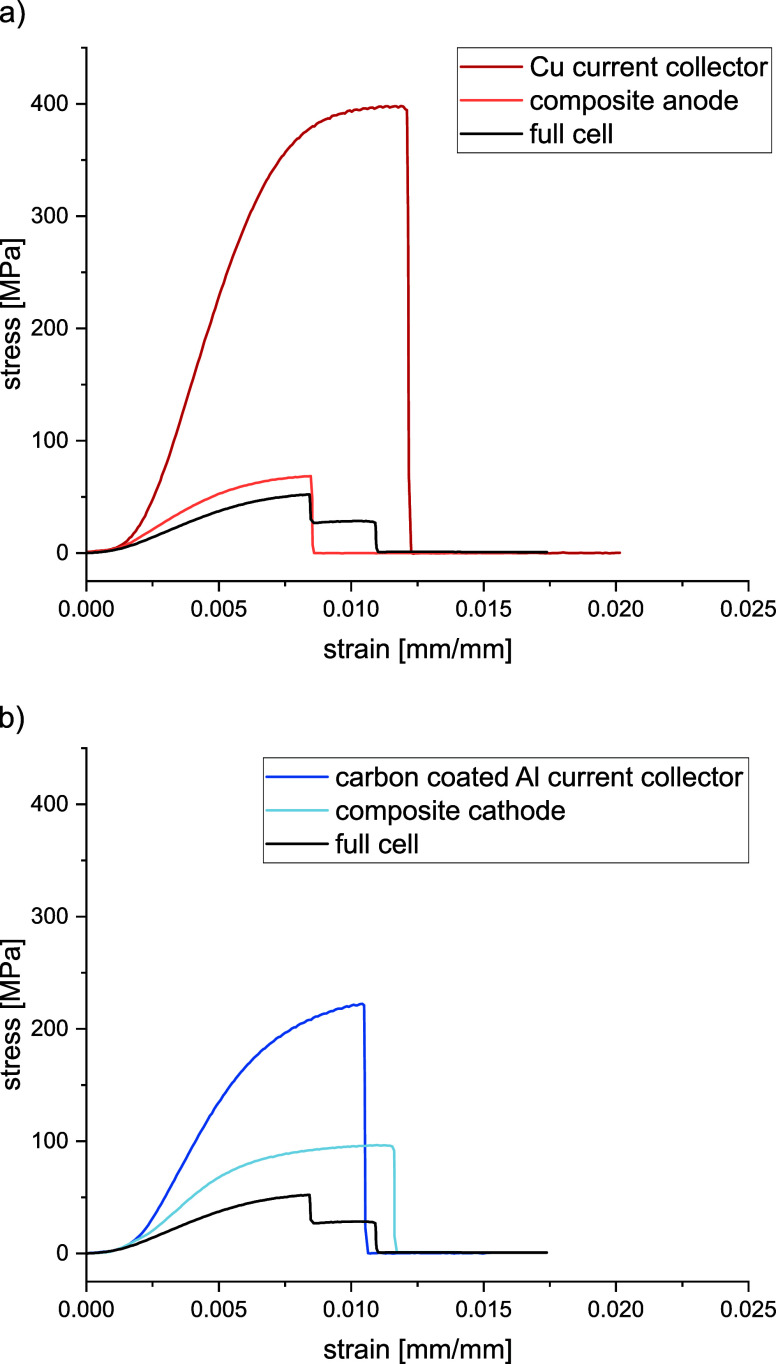
Characteristic stress–strain plots of the Cu current collector, composite anode, and full cell (a), as well as the carbon coated Al current collector, composite cathode, and full cell (b).

To evaluate the multifunctionality of the cell, the mechanical properties were evaluated by tensile testing the assembled full cells as well as their individual components. Dog bone shapes were cut from the prepared electrodes and tested (cf. Figure S8 in the SI). Characteristic stress–strain curves are shown in Figure 10 and Figure S9 in the SI, from which the Young’s moduli in the linear elastic region as well as the tensile strength values were determined. The results are given in Table 1.

**Table 1:** Young’s Moduli of Selected Cell Components as well as the Full Cell after Hot-Calendering

component	Young’s modulus [GPa]	tensile strength [MPa]	fraction of total cell thickness
Cu current collector	74.7 ± 3.3	358.5 ± 21.4	0.068
composite anode	13.9 ± 0.5	65.8 ± 7.1	0.366
carbon-coated Al current collector	42.3 ± 1.4	223.4 ± 9.1	0.114
composite cathode	20.6 ± 1.0	91.2 ± 5.3	0.286
gel electrolyte	0.0184 ± 0.001	3.5 ± 0.3	0.349
full cell	9.9 ± 0.3	49.3 ± 2.1	

The highest Young’s moduli of 74.7 ± 3.3 and 42.3 ± 1.4 GPa were determined for the Cu and Al current collectors, respectively. The addition of the electrochemically active electrode layers reduced the obtained Young’s moduli for the composite electrodes significantly. Especially for the composite anode, the reduction in the Young’s modulus amounts to approximately 81%, whereas for the composite cathode, it is a moderate 51%. These values are in good agreement with the relative thickness of the loading layers with respect to the entire electrode thicknesses. For the composite anode, the loading thickness was around 81%, whereas for the composite cathode, it was 60%. This indicates that the electrochemically active components have a rather low contribution to the mechanical properties of the composite electrodes.

The same trend was observed for the tensile strength which was highest for Cu and Al foils at 358.5 ± 21.4 and 223.4 ± 9.1 MPa, respectively. For the composite electrodes, the values decreased by 81% to 65.8 ± 7.1 MPa for the anode and by 59% to 91.2 ± 5.3 MPa for the cathode, further indicating the low contribution of the electrode loadings. Thus, increasing the mechanical strength of the current collectors by using, e.g., carbon fibers, may significantly increase the mechanical properties of the structural battery and is expected to play a key role in improving the multifunctional efficiency of such batteries.

The assembled full cells were also tested to evaluate their mechanical performance. The Young’s modulus amounts to 9.9 ± 0.3 GPa and the stress–strain curves for the full cells depict a two-step failure. Video imaging showed that the anode side failed first, followed by the cathode side. This aligns with the fact that the composite anode possessed a lower breaking strain and thus failed earlier than the composite cathode. The ultimate tensile strength of the full cells amounts to 49.3 ± 2.1 MPa and thus shows approximately a linear sum of the weighted maximum loads applicable to the composite anode and the composite cathode, respectively (cf. Table 1). It can be concluded that the current collector foils contributed the most to the mechanical properties, and the electrode loadings as well as the gel electrolyte did not significantly add to this. Nevertheless, the Young’s modulus as well as the tensile strength are in the same order of magnitude as it was reported for a structural battery using a carbon fiber anode and a glass fiber separator.^14^

Here, we remark that the general idea of the SOLIFLY as well as the MATISSE project is to integrate batteries into carbon fiber-reinforced polymer (CFRP) laminates. Recently a stack of six battery layups like the ones described in this work were integrated into a CFRP laminate plate and tensile tests were conducted.^58^ This plate exhibited remarkable mechanical properties with a Young’s modulus of 43.2 GPa and an ultimate tensile strength of 270 MPa. This represented a decrease of the Young’s modulus and the tensile strength of 16 and 30%, respectively, compared to a reference CFRP laminate plate without battery cells. Considering that integrating the battery cells into the plate increases the mass of such plates by only 5%, this approach may be a promising solution for multifunctional energy storage.^58^ Since the damage onset of such plates was suggested to be caused by the mechanical failure of the battery cells, detailed investigations and improvements of the mechanical properties of the bare battery layup are desirable.

For further characterization of the mechanical properties, peel tests were conducted to quantify the adhesion between the different layers of the assembled cell and identify the weakest bond. Images of the samples before and after the test as well as a schematic of the test setup are depicted in Figure S10 in the SI. A characteristic peel strength vs displacement plot is depicted in Figure S10f (SI). A rather low force of 0.04 N/mm is enough to detach the cell from the sample holder. The boundary between the Cu current collector and the composite anode loading has been identified as the weakest bond as the peeling of all samples occurred at this interface.

This finding correlates with the tensile tests of the full cells, for which the composite anode was the first to fail, followed by the composite cathode. These results further highlight the importance of adhesion to enable better load transfer across all components of the structural battery. Further development in this regard is assumed to increase the multifunctionality of such devices. Similar reports highlighting the importance of adhesion for other mechanical properties have been reported by Jin et al.^54^

## 4 Conclusions

This study was conducted within the framework of the SOLIFLY and MATISSE projects, both aiming to develop multifunctional energy storage for next-generation aircraft. For this purpose, batteries should be integrated into load-bearing structures consisting of carbon fiber-reinforced polymer (CFRP) laminates. Such laminates are usually handled under ambient conditions and cured at temperatures >100 °C. Furthermore, it was recently shown that the mechanical properties of the battery cell itself influence the mechanical performances of such CFRP laminates with integrated cells.^58^ Thus, a cell chemistry was developed, not only focusing on electrochemical performance but also regarding mechanical properties as well as chemical stability and safety under ambient conditions and elevated temperatures. Specifically, a novel battery has been developed using a gel electrolyte (PVdF-HFP + 3 M LiFSI in PYR13FSI + SiO_2_) and composite electrodes with state-of-the-art battery active materials (NMC811, graphite).

All developed components show high thermal stability up to 150 °C. Beyond this temperature, the gel electrolyte undergoes an exothermic decomposition reaction. H_2_O impurities seem to reduce the thermal stability of FSI^–^-containing components and could not be avoided with the selected processing route inside a dry room. The composite electrodes showed even higher thermal stability, and only some minor signals around 160 °C were observed. The chemical stability of the cell components was evaluated regarding the potential formation of HF upon exposure to moisture. No HF could be detected at ambient conditions (25 °C, 45–55% rel. humidity) for any of the cell components within 24 h from the time of exposure, rendering the developed cell safe for handling under ambient conditions. Furthermore, TGA/FTIR measurements of full cells with temperatures up to 150 °C revealed some evaporation of NMP and H_2_O but no HF was detected.

The electrochemical performance has been evaluated by GCPL for coin and pouch cells. In coin cell format a capacity of about 120 mA h/g_NMC811_ at a C-rate of C/10. In the case of pouch cells, sole stacking of the individual layers led to no considerable electrochemical performance. In contrast, pouch cells which underwent a hot-calendering step after assembly showed a proper performance with 100 mA h/g_NMC811_ at aC-rate of C/10. Thus, good contact between the individual cell layers is crucial. In addition, cells whose components were exposed to ambient air for 2 h prior to the assembly showed no performance decrease compared to cells assembled in the dry room. Half-cell measurements showed that the capacity of NMC811 composite cathodes and graphite composite anodes can be increased from 150 to 170 mA h/g_NMC811_ and from ca. 150 to 340 mA h/g_C_, respectively, by adding 20 μL of the ionic liquid electrolyte. An increased ionic conductivity of the composite electrodes or an improved SEI/CEI formation may lead to this performance increase.

Pouch cells without any additional ionic liquid electrolyte reached an energy density of 370 W h/kg_NMC811_ or 48 W h/kg_Full Cell_. A significant weight penalty comes from the current collectors, which amount to 43% of the cell weight, thus reducing the energy density significantly. However, they also yield mechanical strength, contributing almost entirely to this property, as was verified by tensile testing. The Young’s modulus and tensile strength of the full cell could be measured and amounted to 9.9 ± 0.3 GPa and 49.3 ± 2.1 MPa, respectively. These values are in the same order of magnitude as it was reported for a structural battery using a carbon fiber anode and a glass fiber separator.^14^ Finally, peel tests showed that the weakest bond of the full cell assembly is the interface between the Cu current collector and the composite anode loading. Thus, the cell started to detach always at this boundary. To conclude, this study shows that the developed cells can be handled and integrated safely into CFRP laminates under ambient conditions and curing of such laminate plates may be possible for temperatures <150 °C. Recently, it was shown that such laminate plates with integrated battery cells exhibited a Young’s modulus of 43.2 GPa and an ultimate tensile strength of 270 MPa. Thus, such an approach seems to be promising for multifunctional energy storage since the battery cells increase the plates’ mass by only 5%.^58^

To put these results into perspective, a recent study^59^ reported on the highest Young’s modulus of 76 GPa for a structural battery using carbon fiber as an anode and an LFP-coated carbon fiber as a cathode exhibiting an energy density of 30 W h/kg_Full Cell_.^59^ In general, such approaches usually use an LFP-based cathode and currently do not exceed an energy density of about 45 W h/kg_Full Cell_.^38,59,60^

For the concept presented in this study, further improvements and investigations are needed to achieve the theoretical capacity limits and enable cycling with higher C-rates. Furthermore, improving the mechanical properties is desirable as the battery cell is the weakest link of these multifunctional laminate plates.^58^ One promising route for further development of this system might be the application of alternative current collectors. The overall energy density as well as mechanical properties may be improved by replacing the traditional Cu and Al current collector foils with materials like metalized polymer foils or carbon fibers.

The gel electrolyte can also be further improved; however, a comprehensive understanding of the complex interactions between all components is needed first. Reinforcing the gel electrolyte by the addition of one- and two-dimensional fillers such as fibers and platelets is considered a promising solution. In addition, the amount of filler can be increased, however, at the cost of the ionic conductivity. Therefore, a well-balanced composite needs to be designed to not sacrifice one property over the other. For such Pareto optimizations, computational methods should be applied to reduce the experimental efforts required to find the optimum.

## Supporting Information Available

Photograph of a PVdF-based electrolyte film after drying and before hot-calendering (Figure S1); SEM cross sections of the composite electrodes before and after hot-calendering and densities of composite electrodes (Figure S2); measurement setup for HF-release of the different battery components (Figure S3); measured HF-values after exposure of different cell components to ambient air (Table S1); potential profiles and Coulombic efficiencies of coin cells assembled under dry conditions and after exposure to ambient air for 2 h prior to the assembly (Figure S4); potential profiles of pouch cells assembled with and without a final hot-calendering step (Figure S5); coulombic efficiencies of pouch cells assembled with and without a final hot-calendering step (Figure S6); mass ratio of the different components of a full cell (Figure S7); dog bone shaped composite electrode samples for tensile tests (Figure S8); load-extension curves of tensile tests of composite electrodes (Figure S9); peel test configuration and associated results (Figure S10); impedance spectra of the gel electrolyte (Figure S11) (PDF)
