# Supplementary material for: Multifunctionality and Processability of a Thermoplastic Based Gel Electrolyte Cell for the Realization of Structural Batteries
Source: J Phys Chem C Nanomater Interfaces. 2024 Dec 10;128(50):21317–30. doi: 10.1021/acs.jpcc.4c07301 (PMC11664595; doi:10.1021/acs.jpcc.4c07301)
Supplement: Supplementary file 1 — jp4c07301_si_001.pdf [file jp4c07301_si_001.pdf]

Title:

Multifunctionality and Processability of a Thermoplastic Based Gel Electrolyte Cell for the Realization of Structural Batteries

Authors:

Martin Krammer[1]*, Susan Montes[1], Helmut Kühnelt[2], Qixiang Jiang[3], Daniel Lager[4], Alexander Bismarck[3], Alexander Beutl[1]

*corresponding author

martin.krammer@ait.ac.at

Affiliation:

[1]AIT Austrian Institute of Technology GmbH, Center for Transport Technologies, Battery Technologies, Giefinggasse 2, 1210 Vienna, Austria

[2]AIT Austrian Institute of Technology GmbH, Center for Transport Technologies, Electric Vehicle Technologies, Giefinggasse 2, 1210 Vienna, Austria

[3]Polymer and Composite Engineering (PaCE) Group, Insitute of Materials Chemistry & Research, Faculty of Chemistry, University of Vienna, Währinger Str. 42, 1090 Vienna, Austria

[4]AIT Austrian Institute of Technology GmbH, Center for Energy, Sustainable Thermal Energy Systems, Giefinggasse 2, 1210 Vienna, Austria

A PVdF-based electrolyte film that was fabricated as described for the PVdF-HFP-based electroyte in the main text is shown in Figure S1 after drying. In contrast to the PVdF-HFP-based electroyte, this film exhibits white spots indicating crystallization.

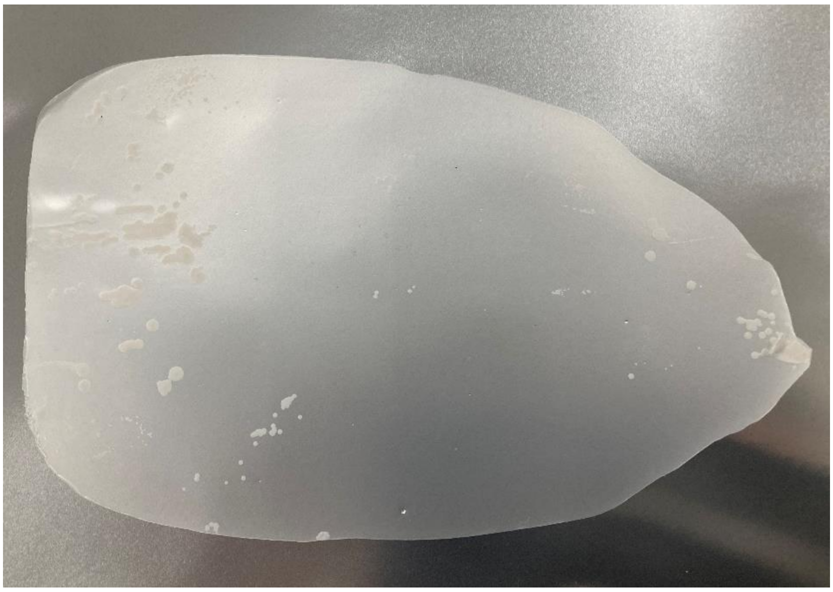

Figure S1: Photograph of a PVdF-based electrolyte film after drying and before hot-calendering.

Figure S2a-d shows the SEM cross sections of the composite electrodes before (a and c) and after hot-calendering. The corresponding densities as well as the expected densities from the rule of mixtures of the components are depicted in Figure S2e.

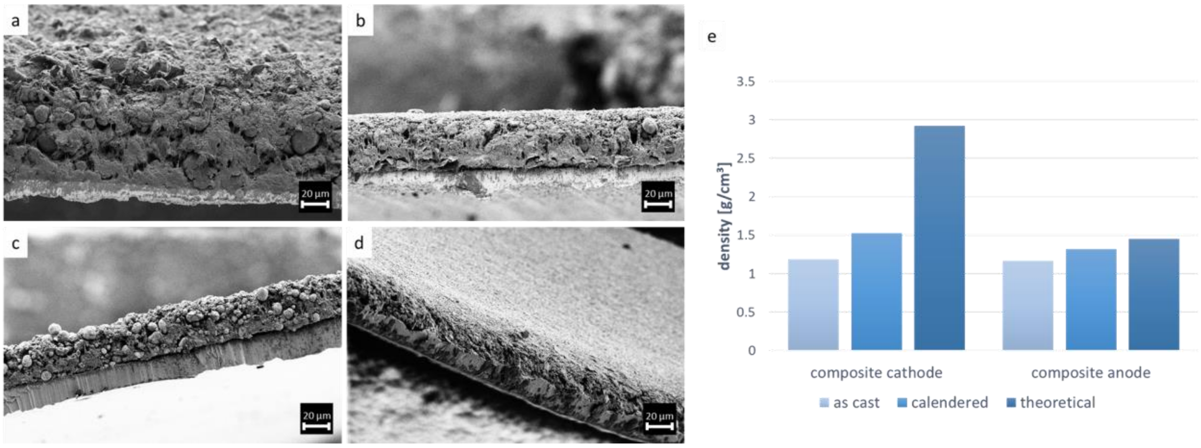

Figure S2: SEM micrographs of the composite anode before (a) and after (b) hot-calendering; SEM micrographs of the composite cathode before (c) and after (d) hot-calendering; e) densities of the as cast and hot-calendered composite electrodes as well as the density expected from the rule of mixtures of the respective components.

All components, i.e., the ionic liquid electrolyte, the gel electrolyte and the composite electrodes have been tested for HF release using a commercial HF detector (measurement range between 0 and 30 ppm with a resolution of 0.1 ppm). For this purpose, all these components were sealed in a desiccator together with the detector and the values were recorded at various times. The corresponding

measurement setup is shown in Figure S3. Even after 24 h, no HF was detected (see Table S1).

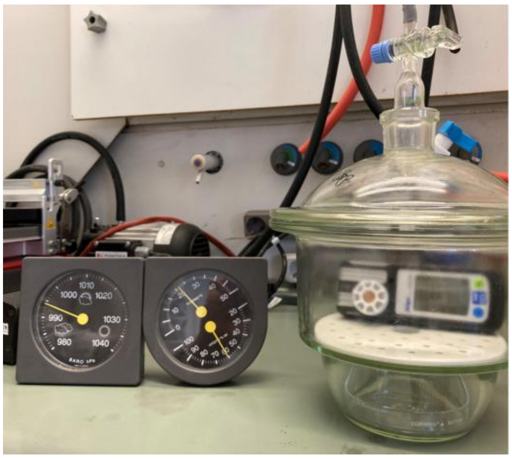

Figure S3: Measurement setup for HF-release of the different battery components. Samples were put together with a HF sensor in a desiccator under ambient conditions and sealed. The HF values were subsequently recorded at different time values.

Table S1: HF-values after exposure of different cell components to ambient air (25°C, 50-60 % rel. humidity).

Sample	HF-sensor readings				
Time	1 h	2 h	5 h	10 h	24 h
3M LiFSI in $PYR_{13}FSI$ (3 g) [ppm]	0.0	0.0	0.0	0.0	0.0
3M LiFSI in $PYR_{13}FSI$ (20 g) [ppm]	0.0	0.0	0.0	0.0	0.0
Cathode (70x100 mm^2) [ppm]	0.0	0.0	0.0	0.0	0.0
Anode (70x100 mm^2) [ppm]	0.0	0.0	0.0	0.0	0.0
Gel electrolyte (70x100 mm^2) [ppm]	0.0	0.0	0.0	0.0	0.0

Figure S4a,b exhibits the potential profiles and coulombic efficiencies of coin cells assembled under dry conditions as well as after exposure to ambient air for 2 h prior to the assembly. Figures S5 and S6 show the potential profiles and coulombic efficiencies of pouch cells, respectively, which were assembled with and without a final hot-calendering step.

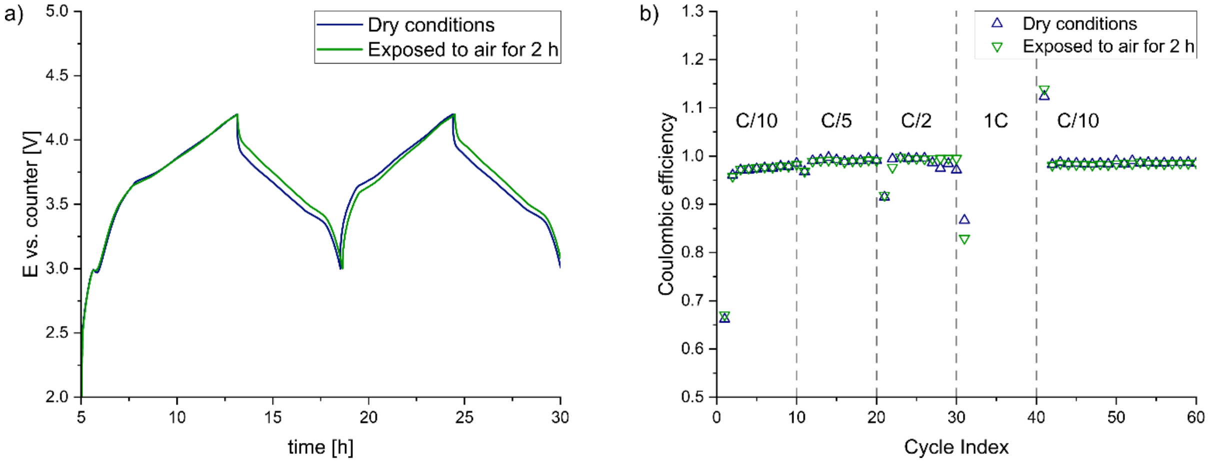

Figure S4: Potential profiles (a) and coulombic efficiencies (b) of full cells in coin cell format assembled under dry conditions as well as after exposure to ambient air.

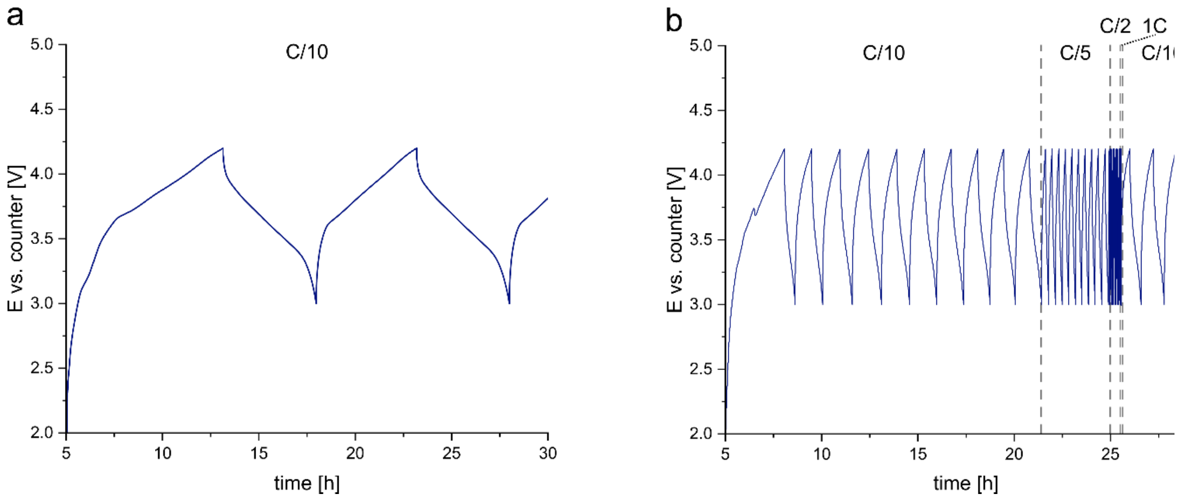

Figure S5: Potential profiles of full cells in pouch cell format with (a) and without (b) a final hot-calendering step, respectively.

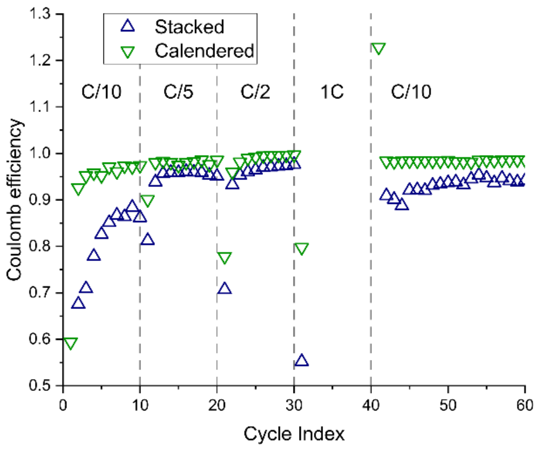

Figure S6: Coulombic efficiencies of full cells in pouch cell format with and without a final hot-calendering step, respectively.

The mass ratio of the different components of a full cell is depicted in a pie chart in Figure S7. The main contribution can be assigned to the current collectors amounting to 43%.

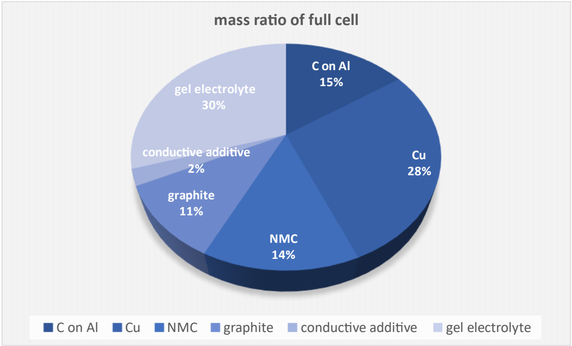

Figure S7: Mass ratio of the different components used for the battery developed in this work.

Figure S8 shows the dog bone shaped composite electrode samples which were used for the tensile tests. Load-extension curves of the corresponding tensile tests can be seen in Figure S9. Furthermore, Figure S10 depicts the peel test configuration and the associated results which were used to determine the adhesion between the different layers of the cell.

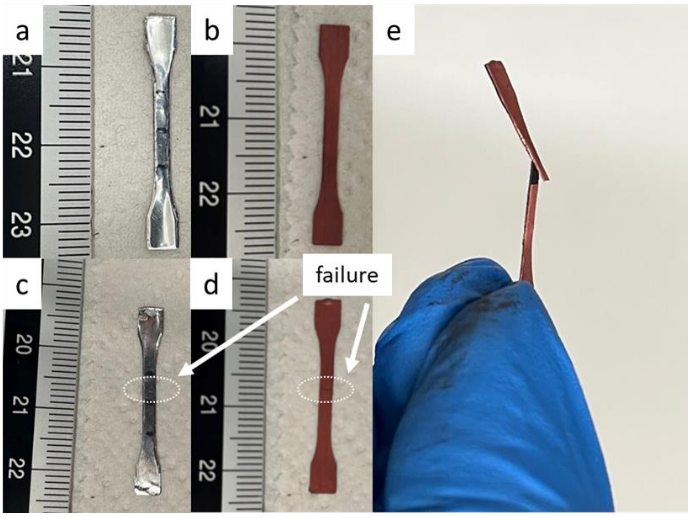

Figure S8: Images of the cathode (a and c) and anode (b,d and e) side of the samples used for tensile testing before (a, b) and after (c-e) the test.

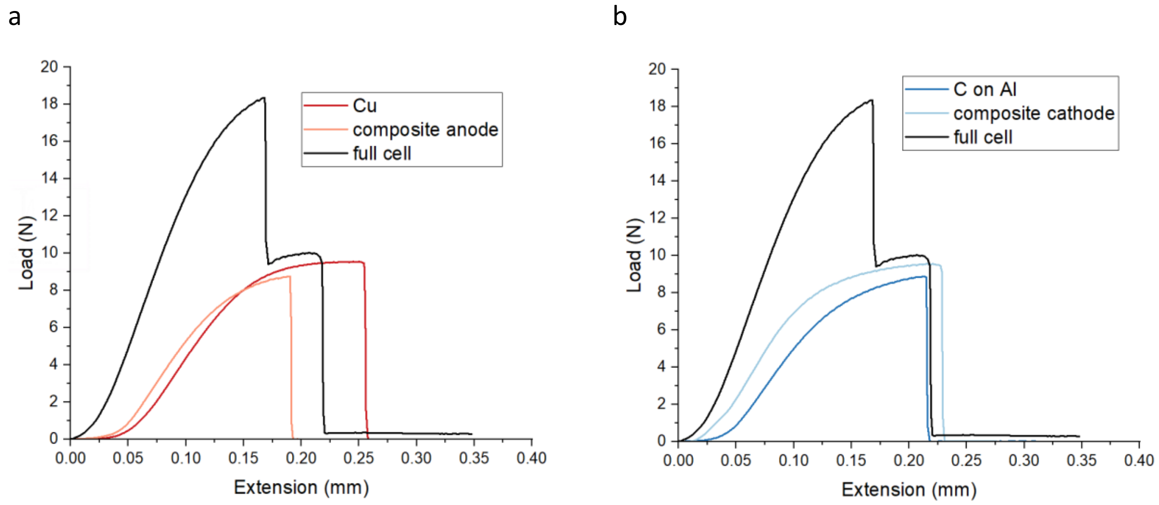

Figure S9: Load vs. extension plots of the Cu current collector, composite anode and full cell (a), and carbon coated Al current collector, composite cathode and full cell (b).

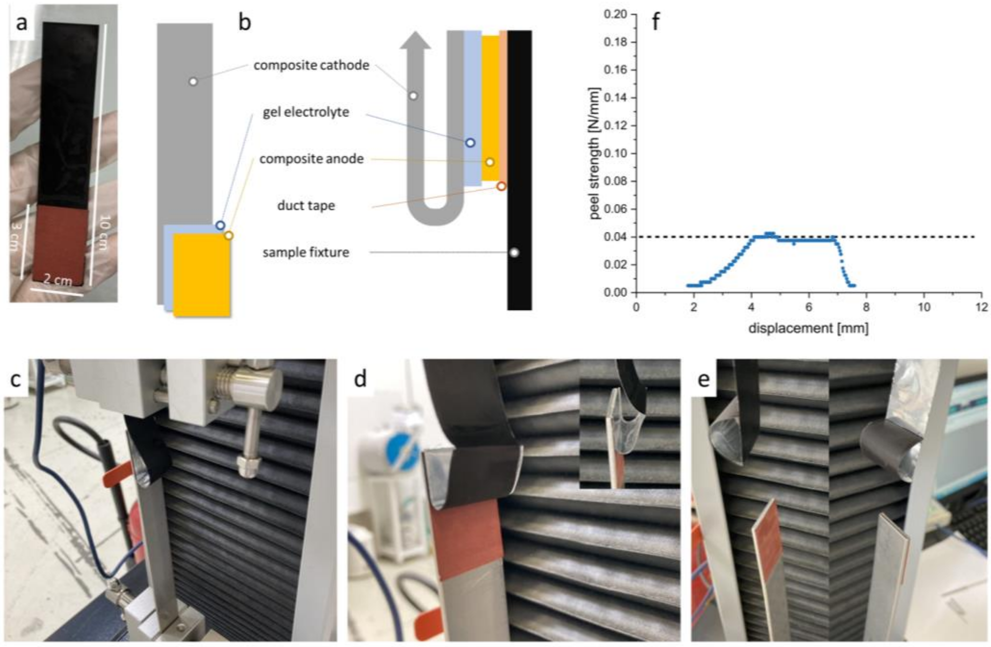

Figure S10: a) Sample used for peeling test; b) schematic of the sample and measuring setup; c)-e) images during the peel test; f) characteristic peel strength vs. displacement plot for full structural cell (black dashed line indicates force of detachment).

Exemplary impedance spectra at 70 °C, 50 °C and 30 °C of the gel electrolyte sandwiched between two ion-blocking stainless steel electrodes are shown in Figure S11. All spectra exhibit an inclined straight line which is typical for the capacitive behavior of ion-blocking electrodes [1]. The intercept with the x-axis gives the ionic resistance of the gel electrolyte which was used for determining the ionic conductivity. As expected, this resistance decreases with increasing temperature. The spectra were fitted with a resistance and a constant phase element (taking into account the nonideal capacitive behavior) connected in series.

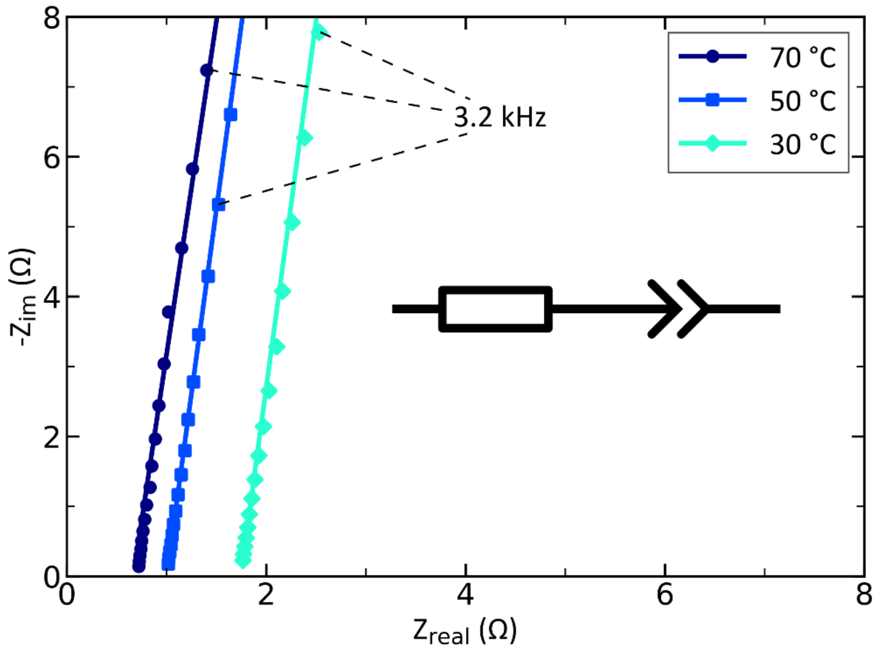

Figure S11: Impedance spectra of the gel electrolyte sandwiched between two ion-blocking stainless steel electrodes at 70 °C, 50 °C and 30 °C. Lines represent fits according to the sketched equivalent circuit.

References

[1] M. Moreno, E. Simonetti, G. B. Appetecchi, M. Carewska, M. Montanino, G.-T. Kim, N. Loeffler, S. Passerini, Ionic liquid electrolytes for safer lithium batteries, Journal of The Electrochemical Society 164.1 (2016): A6026. http://dx.doi.org/10.1149/2.0051701jes
